# Engineering of Metabolic Pathways by Artificial Enzyme Channels

**DOI:** 10.3389/fbioe.2015.00168

**Published:** 2015-10-21

**Authors:** Marlene Pröschel, Rainer Detsch, Aldo R. Boccaccini, Uwe Sonnewald

**Affiliations:** ^1^Department of Biology, Biochemistry Division, Friedrich-Alexander-University Erlangen-Nuremberg, Erlangen, Germany; ^2^Department of Materials Science and Engineering, Institute of Biomaterials, Friedrich-Alexander-University Erlangen-Nuremberg, Erlangen, Germany

**Keywords:** metabolic engineering, matrix-bound enzymes, protein scaffolding, enzyme arrays, metabolic channels, isopeptide-bonding, SpyCatcher/SpyTag, additive manufacturing

## Abstract

Application of industrial enzymes for production of valuable chemical compounds has greatly benefited from recent developments in Systems and Synthetic Biology. Both, *in vivo* and *in vitro* systems have been established, allowing conversion of simple into complex compounds. Metabolic engineering in living cells needs to be balanced which is achieved by controlling gene expression levels, translation, scaffolding, compartmentation, and flux control. *In vitro* applications are often hampered by limited protein stability/half-life and insufficient rates of substrate conversion. To improve stability and catalytic activity, proteins are post-translationally modified and arranged in artificial metabolic channels. Within the review article, we will first discuss the supramolecular organization of enzymes in living systems and second summarize current and future approaches to design artificial metabolic channels by additive manufacturing for the efficient production of desired products.

## Introduction

Living cells are highly dynamic and complex metabolic systems in which most enzymes do not function in isolation but form supramolecular complexes (Jørgensen et al., 2005). By providing spatial and temporal organization of molecules within the cell, these complexes allow optimized substrate channeling and thereby prevent loss of intermediates and improve control and efficiency of catalysis. To mimic supramolecular complexes, several approaches to co-localize functionally related enzymes have been followed. These include scaffolding of enzymes to generate artificial substrate channels (Dueber et al., 2009; Lee et al., 2012). *In vitro* scaffolding of enzymes can be achieved by, i.e., cross-linking, encapsulation, and binding to nucleic acid or protein scaffolds. The latter two options allow the sequential arrangement of enzymes in a correct, programmable, and defined spatial order. Protein-based scaffolding requires specific binding domains for interaction. This bears some problems: only a limited number of high-affinity interaction domains are available, binding efficiency of different domains may not be comparable and interactions are reversible that may result in a short half-life of the artificial channel. To circumvent these problems, covalent linkages between the synthetic scaffold platform and the enzymes to be arranged would be advantageous. In nature, inter- and intramolecular isopeptide bonds are formed to stabilize proteins or to label proteins for proteolysis by ubiquitinylation (Kang and Baker, 2011). By dissecting the mechanism of spontaneous intramolecular isopeptide formation within the CnaB2 domain of the fibronectin-binding protein FbaB from *Streptococcus Pyogenes* (Spy), Howarth and co-workers developed a versatile tool to allow covalent binding of tagged-enzymes to modified macromolecules (Zakeri and Howarth, 2010). This approach can be applied to cell free and possibly even to cellular systems. Besides designing covalent/irreversible or reversible synthetic protein complexes for metabolic engineering, three-dimensional (3-D) printing of enzyme arrays may enable the design of *in vitro* protein channels. These channels do not rely on protein–protein interactions but are based on the sequential printing of individual enzymes. Within the review article, we will describe examples of supramolecular organization in cells, attempts to immobilize and stabilize enzymes for industrial use, and finally summarize current approaches to design artificial metabolic channels by additive manufacturing (AM) for efficient production of valuable chemical products.

## Cellular Proteins are Organized in Supramolecular Structures

**Figure 1:**
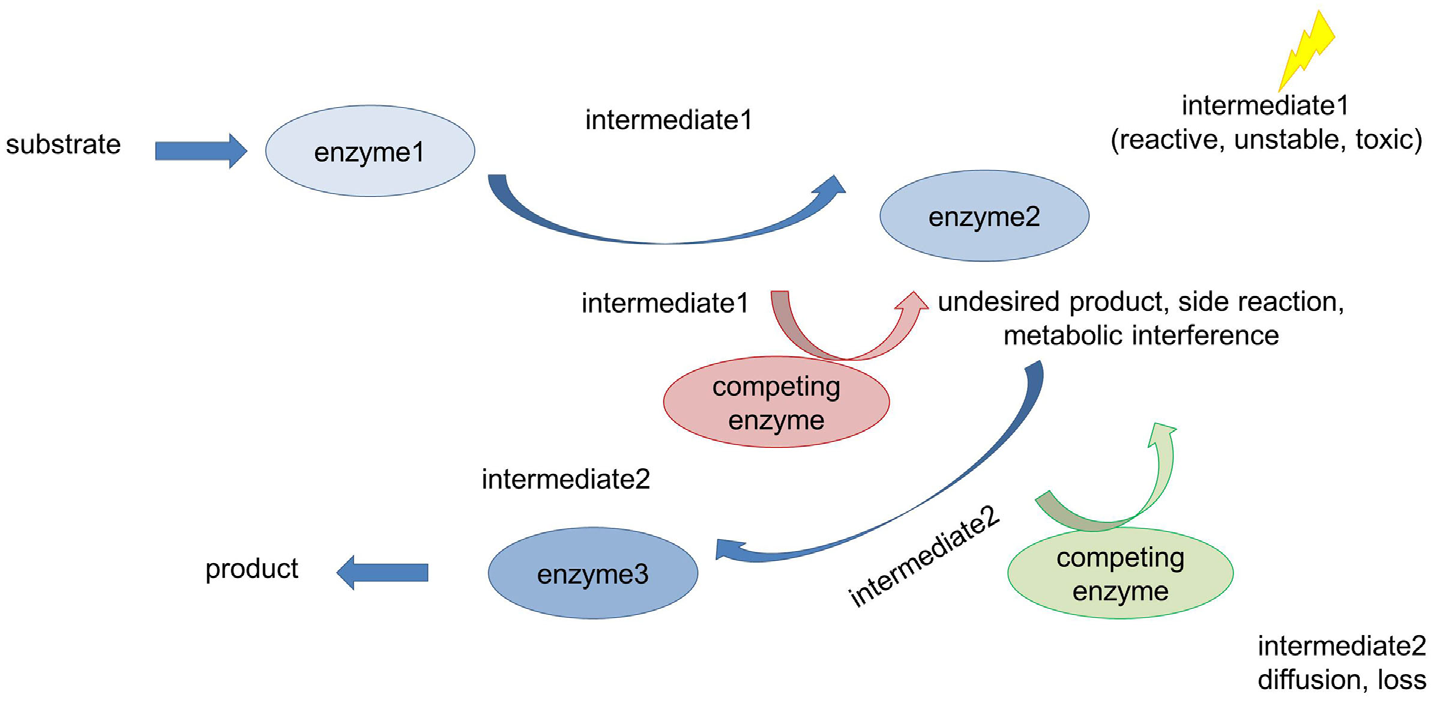
**Metabolic challenges cells have to deal with**. Many metabolic reactions are characterized by low productivity and product yield for the following reasons: accumulation of toxic and/or unstable intermediates, loss of intermediates due to diffusion into the bulk phase (long distances between interacting proteins), and competing pathways (metabolic interference) also leading to undesirable side reactions.

Cellular systems are highly complex and contain high concentrations of macromolecules (Long et al., 2005; Conrado et al., 2008; Good, 2011; Chen and Silver, 2012). Within the cell, these molecules are organized in a temporal and spatial manner allowing the cell to fulfill its many distinct reactions that take place simultaneously (Good, 2011). Coordination and organization of cellular processes is achieved through compartmentation (Chen and Silver, 2012). The need for spatial and temporal organization of proteins in signaling pathways and metabolism is evident when looking at the crowded milieu of macromolecules inside cells and the many complex and competing reactions running concurrently (Sweetlove and Fernie, 2013). In signaling pathways the question arises, how correct interaction partners find each other while avoiding interaction and cross-talk with the wrong ones (Good, 2011). This is important since the correct communication of functionally interacting proteins is a prerequisite for the coordination and regulation of many cellular processes required for appropriate cellular responses to external and internal stimuli (Chen et al., 2014). Strict control and tight regulation of flux through metabolic pathways is of equal importance (Dueber et al., 2009). Metabolic regulation faces many challenges, including avoidance of flux imbalances, slow turnover rates of enzymes, toxic pathway intermediates, and competing metabolic reactions (Figure 1; Conrado et al., 2008; Chen and Silver, 2012; Lee et al., 2012). Consequently, engineering of artificial metabolic pathways in living cells often suffers from low productivity and yield if spatial organization/compartmentation strategies are not included in the engineering concepts (Conrado et al., 2008). To increase the overall cellular efficiency, accuracy, and specificity, nature has evolved compartmentation strategies to control and regulate flux through metabolic and signaling pathways (Chen and Silver, 2012; Conrado et al., 2012).

Intracellular compartmentation can be divided into macro-compartmentation and micro-compartmentation (Sweetlove and Fernie, 2013). Macro-compartmentation refers to the separation of reaction compartments, organelles, by biological membranes. Organelles, which are a hallmark of eukaryotic cells, contain a certain subset of metabolic enzymes that carry out distinct biological reactions. This physical separation of biological reactions increases the overall metabolic efficiency and allows even incompatible or contradictory reactions such as synthesis (anabolism) and degradation (catabolism) or oxidation and reduction to take place within the same cell at the same time. Compartmentation also allows detoxification of toxic pathway intermediates without harming the cell. In peroxisomes, for example, the cytotoxic reactive oxygen species H_2_O_2_ is efficiently degraded by catalase that is specifically present in these organelles. As the amount of catalase in peroxisomes is so high, released H_2_O_2_ is degraded instantly.

**Figure 2:**
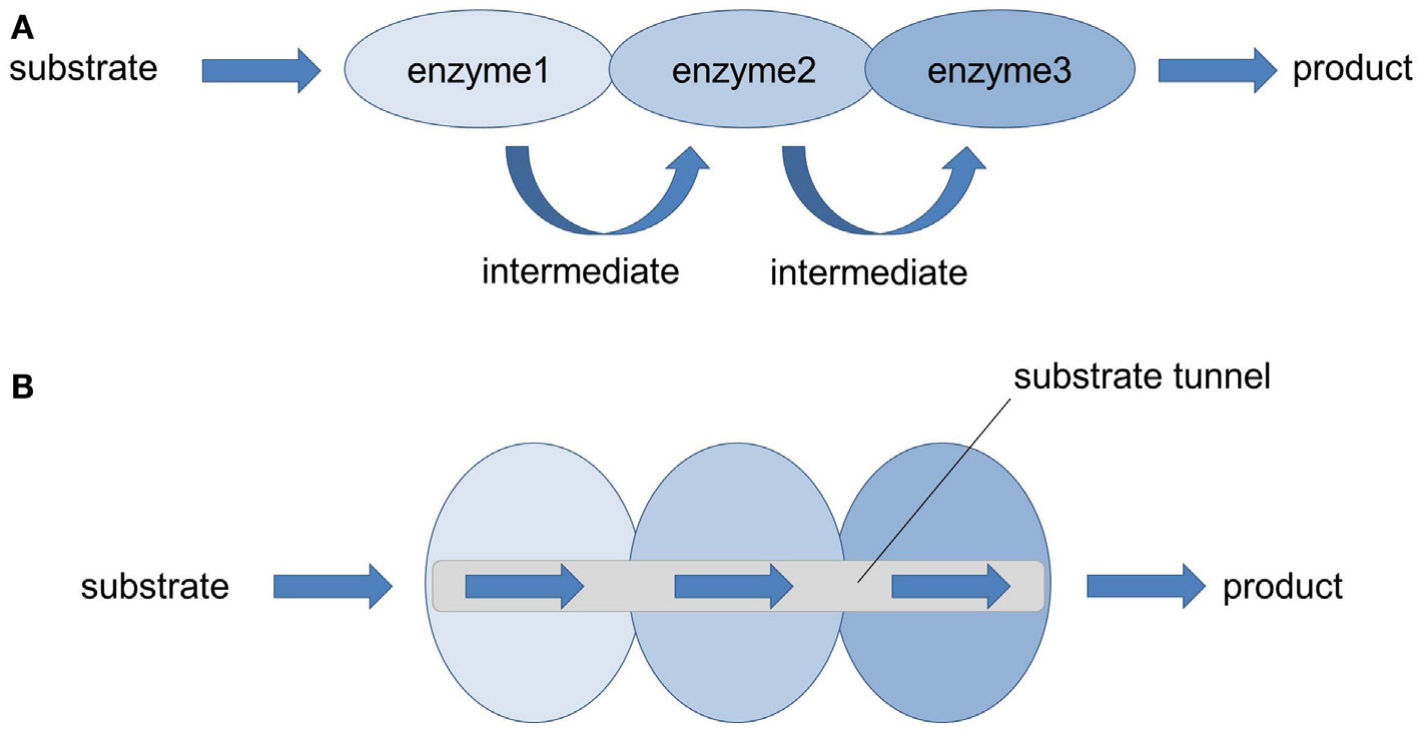
**Multienzyme complexes (= metabolons): nature’s strategy to solve metabolic issues**. **(A)** In nature, many enzymes performing sequential multistep transformation of a substrate are co-localized into multienzyme complexes. Thereby the local concentration of pathway metabolites is increased, the accumulation of pathway intermediates (toxic, reactive, unstable) is limited and the probability of metabolic interference and cross-talk with other cellular components is avoided (Lee et al., 2012). **(B)** Thanks to the enforced proximity of functionally related enzymes, efficient enzyme-to-enzyme channeling through a substrate tunnel can take place, leading to an increased catalytic performance and product yield.

In nature, most cellular multi-cascaded reactions are not catalyzed by free-floating, isolated enzymes but by multienzyme complexes. In these microcompartments, several enzymes form so-called metabolons or metabolic channels overcoming flux imbalances, diffusion and loss of intermediates, and release of toxic intermediates (Figure 2; Conrado et al., 2008; Lee et al., 2012; Jia et al., 2014). The assembly of sequential pathway enzymes into metabolons offers several advantages (Jørgensen et al., 2005; Conrado et al., 2008) when compared to isolated, soluble enzymes. The overall catalytic efficiency is increased because active centers of sequential pathway enzymes are brought into close proximity (enforced proximity) allowing direct transfer of intermediates from one enzyme to the other while avoiding metabolic interference. This phenomenon is called substrate or metabolic channeling and is based on the ordered cascading of subsequent enzymatic steps in which one enzyme produces the substrate of the following enzyme. Thanks to the reduction in diffusion distance and transit time, the effective local concentration of pathway intermediates is higher around the enzyme complex compared to the rest of the cell (Lee et al., 2012). This increase in local concentrations of metabolites prevents unspecific side reactions, favors reaction kinetics, and directs the intracellular flux of metabolites toward the synthesis of the desired product. However, it should be noted that this model is only true for diffusion-limited enzymes (Lee et al., 2012; Sweetlove and Fernie, 2013). In fact, the majority of enzymes are not diffusion-limited which means that the chemistry/conversion process is slower than the diffusion rate. For those non-diffusion-limited enzymes, the most prominent benefits of metabolic channeling are a reduced time to steady state and a better control of reaction specificity and regulation of metabolic branch points (Lee et al., 2012; Sweetlove and Fernie, 2013). Moreover, the release of toxic and/or unstable intermediates into the bulk phase is restricted by such multienzyme complexes that function as pipelines that strictly control the metabolic flux.

There are many examples in a wide range of organisms, including eukaryotes and prokaryotes, where the assembly of different individual sequential enzymes of a metabolic pathway into functional multiprotein complexes increases the overall catalytic efficiency. In the case of primary metabolism, multienzyme complexes are involved in central carbon metabolism (e.g., glycolysis, citric acid cycle), fatty acid oxidation, the Calvin cycle, amino acid biosynthesis (Tryptophan synthesis), the carboxysome, and the proteasome (Conrado et al., 2008). Al-Habori (1995) stated for example that glycolytic enzymes are co-localized on actin filaments to form an active complex. Depending on the energy demand, other authors observed that many of the glycolytic enzymes can be functionally associated with the outer mitochondrial membrane when there is a need for pyruvate to fuel respiration (Giegé et al., 2003; Lunn, 2007; Møller, 2010). This defined intracellular spatial localization of the glycolytic enzymes makes sense as the product pyruvate can directly be transferred into the mitochondria where the next reactions of the central carbon catabolism (citric acid cycle) take place. Reversibility of this spatial organization is important, since pyruvate is an important and central intermediate of several biosynthetic pathways. Several enzymes performing sequential conversion steps in the citric acid cycle are also thought to be associated in a multienzyme complex within the mitochondrial matrix (Barnes and Weitzman, 1986; Lunn, 2007; Jia et al., 2014). The pyruvate dehydrogenase complex that catalyzes the conversion of pyruvate into acetyl-CoA also exhibits an efficient multienzyme structure allowing for substrate channeling and active-site coupling (Smolle and Lindsay, 2006; Jia et al., 2014).

In plants secondary metabolism, biosynthesis of isoprenoids, alkaloids, flavonoids, cyanogenic glucosides (e.g., dhurrin) and phenylpropanoids are examples of the presence/involvement of multienzyme complexes (Winkel, 2004; Jørgensen et al., 2005; Conrado et al., 2008). The phenylpropanoid pathway demands a substantial portion of carbon and energy fixed during photosynthesis. It is organized in a metabolic grid giving rise to a large number of different metabolites (Laursen et al., 2015). Synthesis of the cyanogenic glucoside dhurrin requires seven enzymatic steps starting from tyrosine. In Sorghum bicolor, these reactions are catalyzed by two multifunctional enzymes and one monofunctional enzyme that form a metabolic channel. The importance of this channel became evident in transgenic *Arabidopsis* plants expressing only the two first enzymes of the pathway. As a consequence, the resulting transgenic plants showed significant stunting, most likely caused by accumulation of the toxic pathway intermediate, p-hydroxymandelonitrile. Co-expression of the third enzyme restored normal plant growth and eliminated accumulation of the intermediate.

In fungi, supramolecular enzyme organization can be found in the polyaromatic/shikimate pathway. This pathway includes a multifunctional enzyme known as the AROM complex that has evolved to link five distinct enzymatic activities into a single pentafunctional polypeptide (Conrado et al., 2008). The fungal AROM complex is encoded by a single gene cluster that is expressed in a coordinate manner and also remains associated after synthesis. Interestingly, in *Escherichia coli* and other bacteria, the enzymes are encoded by individual genes distributed throughout the genome (Bachmann, 1983). This precise organization of multiple enzymatic activities on one single peptide, as found in the fungal AROM complex, allows very efficient substrate tunneling between adjacent active sites. Another example of supramolecular enzyme organization in the polyaromatic pathway is the tryptophan synthase. The enzyme is composed of two subunits α and β that assemble as a stable αββα multienzyme complex. Close vicinity of both subunits allows the α subunit to channel the reactive indole intermediate to the β subunit via a hydrophobic, physical tunnel exactly matching indole (Conrado et al., 2008; Dueber et al., 2009). In the case of the tryptophan synthase complex, the active sites are only 25 Å apart from each other leading to the prevention of diffusion of the reactive indole intermediate (Dueber et al., 2009). Thereby the cell is protected and the enzymatic conversion is significantly increased because of the high effective, local concentration of indole (Winkel, 2004; Conrado et al., 2008; Dueber et al., 2009; Chen and Silver, 2012; Lee et al., 2012; Jia et al., 2014).

**Figure 3:**
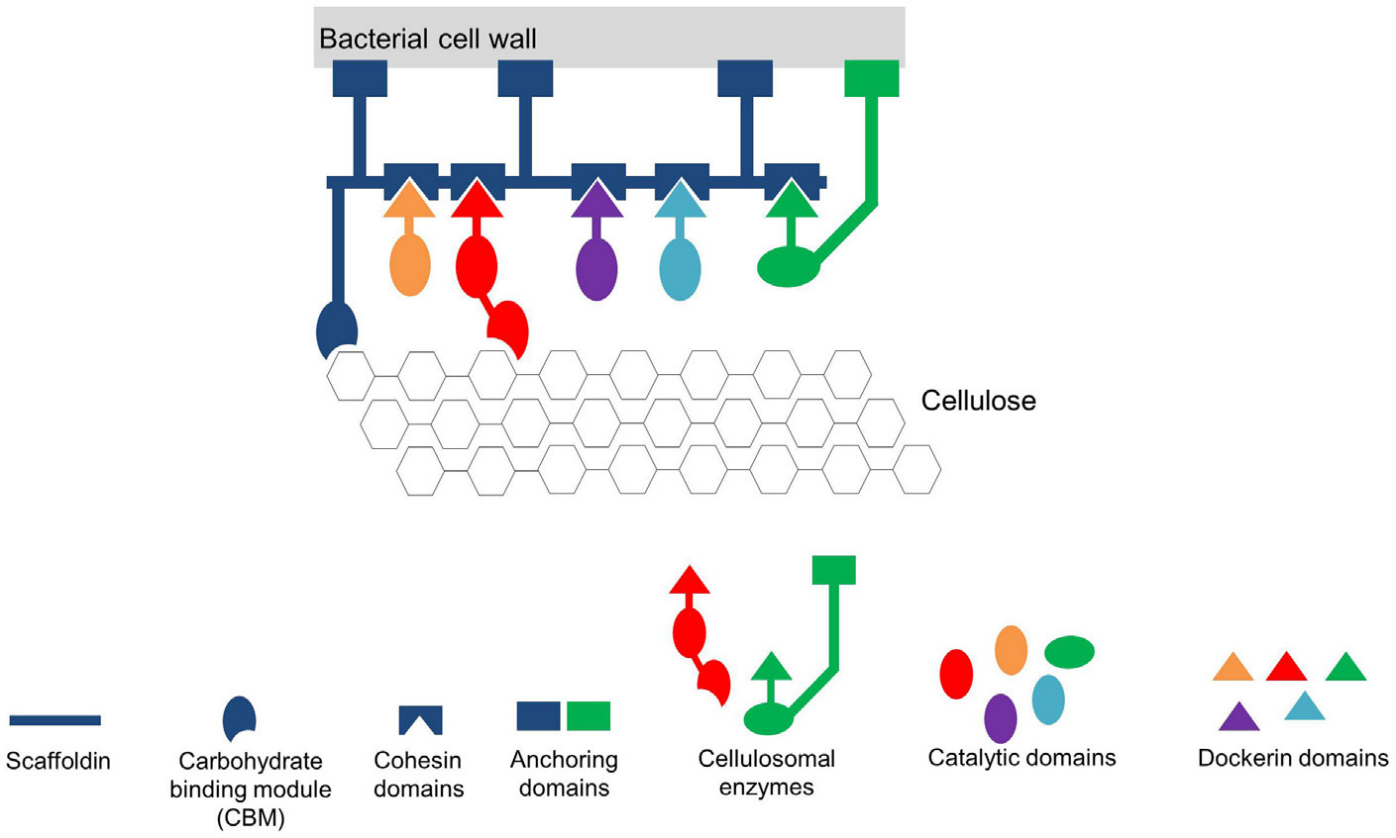
**Simplified scheme of the cellulosome from *Clostridium thermocellum* (adapted from Mazzoli et al., 2012)**. The Cellulosome in this scheme consists of a scaffold protein (Scaffoldin) that is composed of five Cohesin domains. The whole complex is connected to the bacterial cell wall via protein anchors. Each enzyme has its own specific Dockerin domain. By specifically interacting, the Cohesin- and the Dockerin domains form the multienzyme complex in close vicinity to the substrate cellulose. To further increase the affinity of the multienzyme complex toward the substrate the whole scaffold is linked to the substrate via carbohydrate binding modules (CBM).

Bacteria also spatially organize their interior milieu for specialized functions (Chen and Silver, 2012) although lacking membrane-bound organelles as isolated reaction compartments. The interior of prokaryotes can contain various protein-based compartments to physically separate distinct, often critical, enzymatic reactions (Boyle and Silver, 2012). These protein-based compartments are composed of multiple proteins that co-assemble to form thin protein shells and typically encapsulate sequential pathway enzymes (encapsulation). These shells are named bacterial microcompartments (BMCs). The number or amount of encapsulated enzymes is defined by the size of the protein-based compartments. The incorporation of the enzymes into the complex is thought to take place during the assembly of the shell proteins. Small pores (~0.5 nm) on the shells allow the exchange of metabolites according to the size exclusion limit of the pores. Therefore, unrestricted metabolite diffusion across the shell is prevented. Additionally, some of these pores have been shown to be selective (Lee et al., 2012). One example of BMCs found in cyanobacteria and other autotrophic prokaryotes (Chen and Silver, 2012) are carboxysomes (approximately 100 nm in diameter) that are estimated to contain 270 molecules of the key carbon fixation enzyme ribulose-1,5-bisphosphate carboxylase/oxygenase (RuBisCO) (Lee et al., 2012; Chen et al., 2014). In these proteinaceous microcompartments, the local CO_2_ concentration is dramatically increased in the vicinity of RuBisCO preventing the oxygenase reaction of the enzyme and hence increasing its catalytic activity (Lee et al., 2012; Chen et al., 2014). Another example of defined spatial organization to increase metabolic efficiency in prokaryotes is the multienzyme cellulosome complex found in the cellulolytic *Clostridium thermocellum* (Chen and Silver, 2012). Due to the specific interaction of the complementary protein domains Dockerin and Cohesin, many different enzymes required for the degradation of plant cell walls are organized as extracellular nanomachinery on a scaffold on the cell surface (Figure 3; Lytle and Wu, 1998; Bayer et al., 2004; Pinheiro et al., 2009; Mazzoli et al., 2012). The organization and co-localization of the different hydrolytic enzymes and the close proximity to the substrate provides an efficient synergistic strategy to degrade cellulose and hemicellulose (Gefen et al., 2012).

In mammalian cells, the purinosome catalyzes the conversion of phosphoribosyl pyrophosphate (PRPP) to inosine monophosphate (IMP). This conversion requires six enzymes that are co-localized on microtubules to form an efficient metabolon. Assembly and disassembly are highly regulated and link the rate of *de novo* purine synthesis to the cellular purine nucleotide pool (DeLisa and Conrado, 2009). The purinosome complex is formed upon depletion of purines. The complex is associated with microtubules and stabilized by molecular chaperones, including HSP70 and HSP90. Accumulation of purines induces the disassembly of the complex. This process involves reversible protein phosphorylation by protein kinase CK2 (for review, see Laursen et al., 2015). Assembly and disassembly of purinosomes are remarkable examples of the dynamic regulation of multienzyme complexes which is of outmost importance to tightly coordinate metabolic flux through metabolic channels (Conrado et al., 2008; DeLisa and Conrado, 2009; Møller, 2010).

If not encoded by a single gene cluster (i.e., the AROM complex in fungi), the described supramolecular enzyme complexes assemble post-translationally into rather stable or transient complexes. Transient complexes allow dynamic responses to intra- (e.g., metabolic demands) and extracellular stimuli or signals (e.g., abiotic and biotic challenges) which is important for fine-tuning metabolism according to the state/demand of the cell or organism (Conrado et al., 2008; DeLisa and Conrado, 2009; Møller, 2010; Sweetlove and Fernie, 2013). Transient or dynamic metabolons are also called “functioning-dependent structures” (Møller, 2010). This micro-compartmentation can be achieved in different ways. One strategy of living cells is to co-localize interacting proteins/enzymes by anchoring them on membranes or on cytoskeleton structures (actin, microtubules). Alternatively, multienzyme complexes are organized by scaffold proteins (Good, 2011). The latter strategy is found in some metabolic pathways such as the cellulosomes but occurs frequently in signal cascades. In yeast, for example, the Ste5 scaffold protein organizes the interaction between Fus3 (MAPK), Ste7 (MAPKK), and Ste11 (MAPKKK) and is essential for mating. In mammalian cells, the Kinase suppressor of Ras (KSR) functions as a scaffold in the Ras–Raf–MEK–MAPK pathway (Roy et al., 2002) and the PSD-95 synaptic scaffold is crucial for the organization of neuronal synapses controlling the neurotransmitter receptor density (Good, 2011). Scaffold proteins are defined as extremely diverse proteins that coordinate the physical assembly of individual partner molecules (Good, 2011). Scaffold proteins, composed of multiple modular interaction domains (for example, protein–protein interaction domains) or motifs (Good, 2011), form flexible platforms where other proteins or relevant molecular components of a specific pathway can bind to. Thereby, the interaction partners are co-localized in a modular manner. Signaling pathways, where cascaded enzyme reactions often take place, especially benefit from this subcellular, spatial organization. Scaffolding strategy supports specificity, accuracy, and efficiency of signal transduction pathways, by enforcing proximity of the correct interaction partners whereas incorrect interaction partners are excluded (Good, 2011).

## Synthetic Multienzyme Complex Formation to Mimic Nature’s Strategy to Increase Metabolic and Signaling Efficiency

In metabolic engineering where a natural endogenous biosynthetic pathway is manipulated to increase productivity and yield of a valuable molecule (Sonnewald, 2003; Capell and Christou, 2004; Na et al., 2010; Chen and Silver, 2012), several challenges have to be overcome. One is the expression level of heterologous enzymes. In bacterial systems, expression levels are often much higher compared to endogenous enzymes. This can lead to cellular stress responses, for example, due to the huge amount of proteins produced that are often even unfolded or misfolded, flux imbalances coupled with unpredictable and non-controllable metabolic changes and high energy and molecule (amino acids, nucleotides) consumption. An additional challenge is to balance expression of consecutive enzymes (Na et al., 2010). If the reactions are not balanced in a manipulated pathway, toxic intermediates are likely to accumulate which can lead to death of the expression host (Dueber et al., 2009). With nature’s strategies to increase metabolic efficiency in mind, metabolic engineers are trying to engineer artificial multienzyme complexes, where the enzymes performing consecutive reactions are spatially organized (directed enzyme organization). The idea is to co-localize functional enzymes into complexes. Due to the enforced proximity of the enzyme active sites and the formation of enzyme microdomains built as a consequence of coclustering of multiple enzymes into higher aggregates, catalytic efficiency and metabolic pathway performance are improved (Sweetlove and Fernie, 2013; Castellana et al., 2014). Overall, pathway balancing involves several layers, including DNA copy number, transcriptional and translational regulation, scaffolding and compartmentation, as well as inclusion of metabolic sensors balancing the flux through synthetic pathways (Boyle and Silver, 2012; Jones et al., 2015). In addition to balancing protein amount and organization, enzyme engineering allows to improve activity, selectivity, and stability of enzymes (Otte and Hauer, 2015). Several approaches can be followed to stabilize enzymes. One promising approach involves cyclization of enzymes which has been achieved by using the split intein (Zhao et al., 2010) or SpyTag/SpyCatcher system (Schoene et al., 2014).

## Non-Programmable Matrix-Bound Enzyme Complexes

One strategy of engineering efficient multienzyme complexes that mimic those found in nature is to co-immobilize multiple enzymes of a sequential/cascaded pathway on the same carrier or on the same matrix. Early studies on immobilized enzymes clearly demonstrated that tethering of enzymes to particles significantly improves product formation. Comparing the activity of three successive enzymes, β-galactosidase, hexokinase, and glucose-6-phosphate-dehydrogenase, either matrix-bound or soluble, Mattiasson and Mosbach (1971) observed a faster conversion of the substrate when the sequential enzymes are co-localized on the same particle. Coupling was achieved by the CNBr method yielding covalently bound enzymes. Obvious disadvantages of the system include (i) spatial arrangement of enzymes is impossible and (ii) chemical cross-linking bears the risk of losing enzyme activity because chemical cross-linking is random and can also affect amino acid residues in the active center of the enzyme. The group of Mallapragada (Jia et al., 2013) sequentially co-localized the two model enzymes glucose oxidase (GOX) and horseradish peroxidase (HRP) on dual-functionalized polystyrene nanoparticles. The nanoparticles had been functionalized with carboxyl groups which have partially been modified using biotin hydrazide resulting in biotinylated carboxyl-polystyrene nanoparticles. This dual-functionalization allowed the use of different attachment strategies for each enzyme to better control the relative amounts of the enzymes on the nanoparticle. The streptavidin-tagged HRP was attached to the nanoparticles via the high-affinity biotin–streptavidin interaction. The unmodified carboxyl groups on the nanoparticles were used to covalently attach GOX by amide bond formation between the reactive carboxyl groups on the nanoparticle and amino groups of the enzyme. Immobilized enzymes retained their enzymatic activity that was comparable to free enzymes. Interestingly, sequential co-localization of GOX and HRP resulted in a twofold enhancement of the overall product conversion rate compared to the free enzymes and a mixture of individual immobilized enzymes on nanoparticles (Jia et al., 2013).

In several studies, immobilization of enzymes caused positive catalytic/kinetic effects and at the same time resulted in the stabilization of various enzymes (Sheldon, 2007; Garcia-Galan et al., 2011; Homaei et al., 2013; Guzik et al., 2014). For industrial applications, immobilized enzymes have additional advantages. They can be reused over multiple cycles and the enzymes are sequestered from the product stream. These properties improve industrial processes and first applications of immobilized enzymes include glucose isomerase for high fructose corn syrup, lipase for biodiesel production from triacylglycerides or thermolysin for aspartame synthesis (DiCosimo et al., 2013). Compared to the conventional fossil fuel-based chemistry, biomanufacturing offers many advantages, including biocompatibility and sustainability that leads to massive growth rates of the world market for industrial enzymes.

## Synthetic Compartmentation by Protein Encapsulation

Besides sequestration or tethering of enzymes using different matrices, encapsulation of proteins into semi-permeable compartments allows the physical separation of different metabolic reactions leading to an increase in pathway efficiency. As discussed above, bacteria encode proteinaceous shells, BMCs, in which functionally related enzymes are sequestered. Up to now BMC-shell encoding genes have been found in over 400 different sequenced bacterial genomes (Choudhary et al., 2012). The proteins form polyhedral structures similar to virus-like particles. Co-expression of recombinant shell proteins and selected proteins of interest (e.g., pathway enzymes), fused to shell-targeting signal peptides, in *E. coli* cells, leads to the functional compartmentation of heterologous enzymes in recombinant shells (Choudhary et al., 2012). In addition to bacterial shell proteins, also viral capsid proteins have been used as a tool for compartmentalizing engineered pathways (Chen et al., 2014). Co-expression of two or three enzymes fused by linker sequence to form one multifunctional protein with the bacteriophage P22 capsid protein allowed the design of a synthetic metabolon (Patterson et al., 2014). To improve the suitability of capsid and cargo proteins, several modifications have been tested. By adding positively charged peptides to capsid proteins and negatively charged peptides to cargo proteins, binding could be improved via enhanced electrostatic forces (Chen et al., 2014).

## Programmable and Reversible Scaffolding

**Figure 4:**
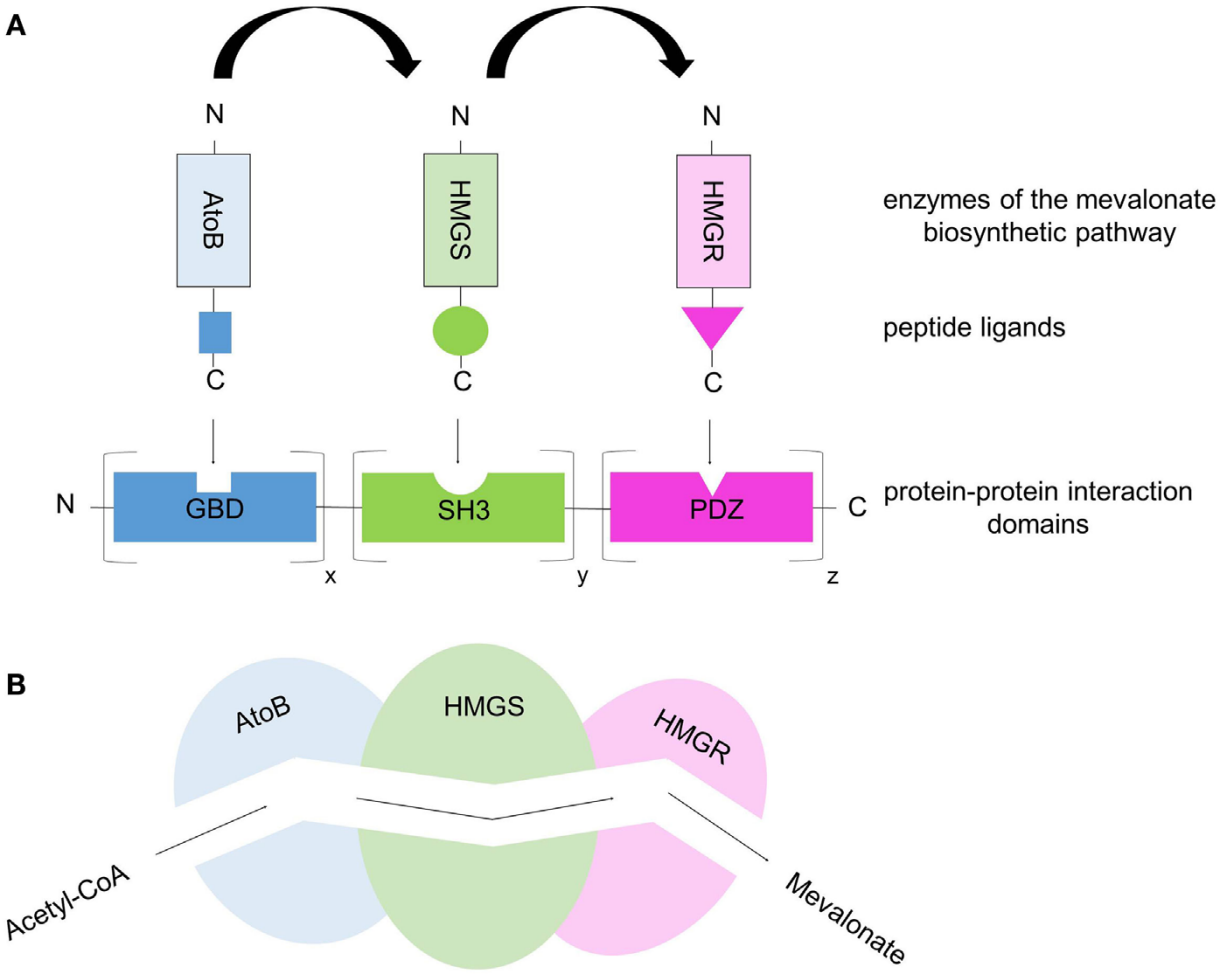
**Protein-scaffold-based multienzyme complex offers a synthetic metabolic pipeline that allows substrate channeling**. **(A)** The three enzymes of the mevalonate biosynthetic pathway (acetoacetyl-CoA thiolase, AtoB; hydroxy-methylglutaryl-CoA synthase, HMGS; hydroxy-methylglutaryl-CoA reductase, HMGR) were co-localized on a synthetic protein-scaffold via high-affinity interactions between protein domains and their specific, cognate peptide ligands. The protein scaffold consists of various repeats of well-characterized, metazoan protein domains (SH3, GBD, PDZ) and the corresponding peptide ligands are fused to the enzymes. By co-expressing the synthetic scaffold and the enzyme–peptide ligand fusions in *E.coli* cells, the three enzymes are targeted to the scaffold building a multienzyme complex. Through the variation of the binding domain repeats the ratio/stoichiometry of enzymes can be controlled. **(B)** Simplified scheme of substrate channeling through a tunnel/metabolic pipeline connecting the active sites of the co-localized enzymes.

Besides the co-immobilization strategy of enzymes on carriers to build efficient synthetic multiprotein complexes, various genetic modules (proteins, nucleic acids) have been described to function as building blocks for generating programmable, modular scaffolds on which enzymes can specifically be co-localized in a spatially ordered manner by simple tethering mechanisms. Specific protein–protein, DNA–DNA/RNA–RNA, and DNA–protein/RNA–protein interactions have been used to co-localize metabolic enzymes to improve pathway flux. Dueber et al. (2009) designed synthetic protein scaffolds out of known, well-characterized, and widespread protein–protein interaction domains from metazoan signaling proteins (SH3-, PDZ-, and GBD binding domain). By fusing enzymes of the mevalonate biosynthetic pathway (AtoB, HMGS, and HMGR) to the respective peptide ligands (SH3-, PDZ-, and GBD-ligand), the authors generated a modular genetically encoded scaffold system on which the enzymes can be co-localized in a programmable and defined manner (Figure 4). This scaffold approach is based on simple, specific high-affinity interactions between protein binding domains and their cognate, specific peptide ligands. In addition to the close proximity of the enzymes co-immobilized on the protein scaffold and the substrate channeling resulting from this, the reaction efficiency and the overall production rate/product yield can be further improved by varying the number of protein binding domain repeats in the scaffold taking into account the kinetic properties of the individual enzymes. Therefore, potential enzymatic bottlenecks, e.g., caused by low Kcat values or binding affinities (K_M_ values), can be compensated by simply increasing the amount of “weak” enzymes with slow turnover rates. When the optimal scaffold stoichiometry (optimal enzyme ratio) is used, the conversion from acetyl-CoA to mevalonate performed by the three sequential enzymes AtoB, HMGS, and HMGR produced a 77-fold higher level of the product mevalonate compared to that of the un-scaffolded pathway (Dueber et al., 2009; Lee et al., 2012; Chen et al., 2014). This demonstrates once again that the spatial organization of enzymes into functional complexes allowing effective substrate channeling increases the overall metabolic efficiency because the local concentrations of metabolic intermediates are increased while their accumulation to toxic levels is prevented (Dueber et al., 2009). The same modular protein-based scaffold strategy was applied to the three-enzyme glucaric acid pathway, where glucaric acid is produced from glucose. Compared to the free enzymes, glucaric acid levels have been improved fivefold by scaffolding in *E. coli* cells (Moon et al., 2010; Lee et al., 2012). Another example of the use of synthetic protein scaffolds to increase pathway flux is resveratrol biosynthesis in yeast cells (Wang and Yu, 2012). Here, the authors scaffolded two enzymes, 4-coumarate: CoA ligase and stilbene synthase and achieved a fivefold increase in resveratrol synthesis compared to the un-scaffolded control. While the above given examples demonstrate the power of protein scaffolding using protein–protein interaction domains from metazoan signal transduction pathways, these domains may not be applicable in all systems. They may misfold or aggregate and cross-talk between engineered scaffolds with native signaling molecules may occur. The potential risk of cross-talk is dependent on the organism and may be neglectable for *E. coli* cells in which the described domains are not present (Dueber et al., 2009). To circumvent unintended perturbations of signaling pathways of the expression host, minimized synthetic domains or alternatives are required. One alternative are interaction domains derived from the bacterial cellulosomes. Based on the modular architecture of bacterial cellulosomes (discussed above), complementary protein modules of Cohesin and Dockerin can be used to generate artificial multienzyme complexes. Any enzyme of interest can be genetically fused to Dockerin domains. A scaffold consisting of various Cohesin domains leads to the targeting of the enzymes in a defined spatial orientation due to the specific protein–protein interaction between Cohesin domains and their cognate Dockerin domains. Thereby the so-called “Designer-Cellulosomes” can be generated.

An alternative to protein scaffolds are nucleic acid-based (DNA or RNA) scaffolds. DNA and RNA molecules represent suitable modular tools for the specific programmable spatial organization of pathway enzymes as they provide specific interactions by either hybridization (base pair complementarity, DNA–DNA/RNA–RNA binding) or protein binding sequences specific for engineered zinc-finger or TALE proteins (DNA/RNA-protein binding). Conrado et al. (2012) used a configurable DNA-based scaffold to spatially arrange multiple pathway enzymes in a distinct order in the cytoplasm of *E. coli*. To specifically target pathway enzymes to the plasmid DNA-based scaffold consisting of multiple unique zinc-finger binding sites, the enzymes of interest were genetically fused to corresponding zinc-finger domains that specifically bind the DNA sequences present in the engineered scaffold with high affinity. Increased overall production rates due to the DNA scaffold-mediated enzyme co-assembly could be observed when enzymes of the resveratrol biosynthesis and the mevalonate synthesis were used (Conrado et al., 2012).

Another example of a DNA-assembled artificial multienzyme complex made use of luciferase and oxidoreductase catalyzing two consecutive reactions of flavin mononucleotide reduction and aldehyde oxidation (Niemeyer et al., 2002; Müller and Niemeyer, 2008). DNA hybridization of complementary ssDNA oligonucleotides in combination with the high-affinity biotin–streptavidin protein interaction was used to build the bienzymatic complex. Biotinylated enzymes were linked to covalent ssDNA-streptavidin conjugates resulting in enzyme–DNA conjugates due to the specific and very strong high affine, but non-covalent interaction between biotin and streptavidin. As single-stranded DNA carrier strands, containing complementary regions to the DNA oligomers found in the enzyme–DNA conjugates, were previously immobilized on a surface (microtiterplate) via the biotin–streptavidin interaction, co-immobilization and therefore multienzyme complex formation of the enzymes takes place by specific DNA–DNA hybridization (complementary base pairing) (Niemeyer et al., 2002). To explore the proximity effect caused by the spatial arrangement of the two enzymes, activity assays were performed. Results indicate that the enforced spatial proximity of the enzymes luciferase and oxidoreductase increases the overall enzymatic activity of the bienzyme complex in a scaffold architecture dependent manner (Niemeyer et al., 2002). Almost the same DNA-scaffold approach was used by Müller and Niemeyer (2008). The authors reported on the DNA-directed assembly of GOX and HRP. Both enzymes were used because they are a suitable reporter system for which the kinetic rates and the output can easily be measured as the two-step reaction performed by these enzymes produces a highly fluorescent dye (Resorufin) when providing Amplex Red. Catalytic efficiency could be measured by monitoring the fluorescence emission. In this study, the authors could show that the efficiency of the two DNA–enzyme conjugates was dependent on the position and steric parameters (Müller and Niemeyer, 2008).

RNA molecules also provide a modular tool for scaffold approaches where multiple pathway enzymes can be targeted and thereby spatially organized. Delebecque et al. (2011) used RNA aptamers (= short single-stranded oligonucleotides) to create 1D and 2D scaffolds. These scaffolds were used for the spatial organization of two bacterial enzymes required for hydrogen production. Similar to protein- and DNA-scaffolds, RNA-based scaffolds increased the rate of substrate conversion and product yield (Delebecque et al., 2011; Conrado et al., 2012). While proteins and especially large protein fusions often tend to misfold or aggregate, nucleic acids (DNA and RNA) have highly predictable local structures, and enzymes can be arranged into a given and programmable order by changing the distance between the protein binding sites (Conrado et al., 2012; Chen et al., 2014). Furthermore, DNA and RNA can easily be produced, fold into various structures (high-order assemblies), can have different lengths, and can consist of flexible numbers of repetitive scaffold units (Chen et al., 2014). For protein-based scaffolds, the addition of only short peptide ligands to each pathway enzyme is recommended to ensure the correct folding and therefore the function of the enzymes (Lee et al., 2012). In addition of course the fusion of, for example, zinc-finger domains to enzymes is critical, therefore an impact on the enzyme stability and activity cannot be excluded. Consequently, all modifications performed on the enzymes as well as on the modules (proteins, nucleic acids) to design the synthetic scaffold have to be optimized.

Besides all the advantages such as modularity and specificity, a big disadvantage of these scaffolds based on high affine protein–protein interaction domains is that the interactions are reversible depending on the different dissociation constants of the binding domain/peptide ligand pairs and they do not resist forces or boiling. Furthermore, not so many protein–protein or protein–peptide interactions are known that have low (nM) dissociation constants which is a measure for the affinity. In addition, each specific pair has its own dissociation constant and often they are not comparable with each other. This influences the targeting of the ligand-fused enzymes to the scaffold out of the protein binding domains. The different affinities complicate the specific and exact targeting of single enzymes to the scaffold in a defined ratio. There is also the risk of uncontrolled dissociation of the binding domain/peptide ligand pairs that are reversible and do not resist forces as the interaction is not covalent. Therefore, it would be desirable to create covalent linkages between proteins that are specific, formed under a wide range of conditions and which require fusion of small peptides only.

## Programmable and Irreversible Scaffolding of Enzymes

Inspiration for the design of intermolecular covalent linkages between proteins originates from the analysis of pili formation in Gram-positive bacteria. Pili of Spy are composed of three subunits, Spy0125, Spy0128, and Spy0130 and are required for adhesion to host cells. Besides the well-known disulfide bridges between two cysteine residues, structural analysis of these and other bacterial surface proteins uncovered several additional covalent intramolecular bonds, including isopeptide bonds between lysine and asparagine or lysine and aspartate (Kang et al., 2014), ester bonds between threonine and glutamine (Kwon et al., 2014) or thioesters between cysteine and glutamine (Pointon et al., 2010; Walden et al., 2015).

**Figure 5:**
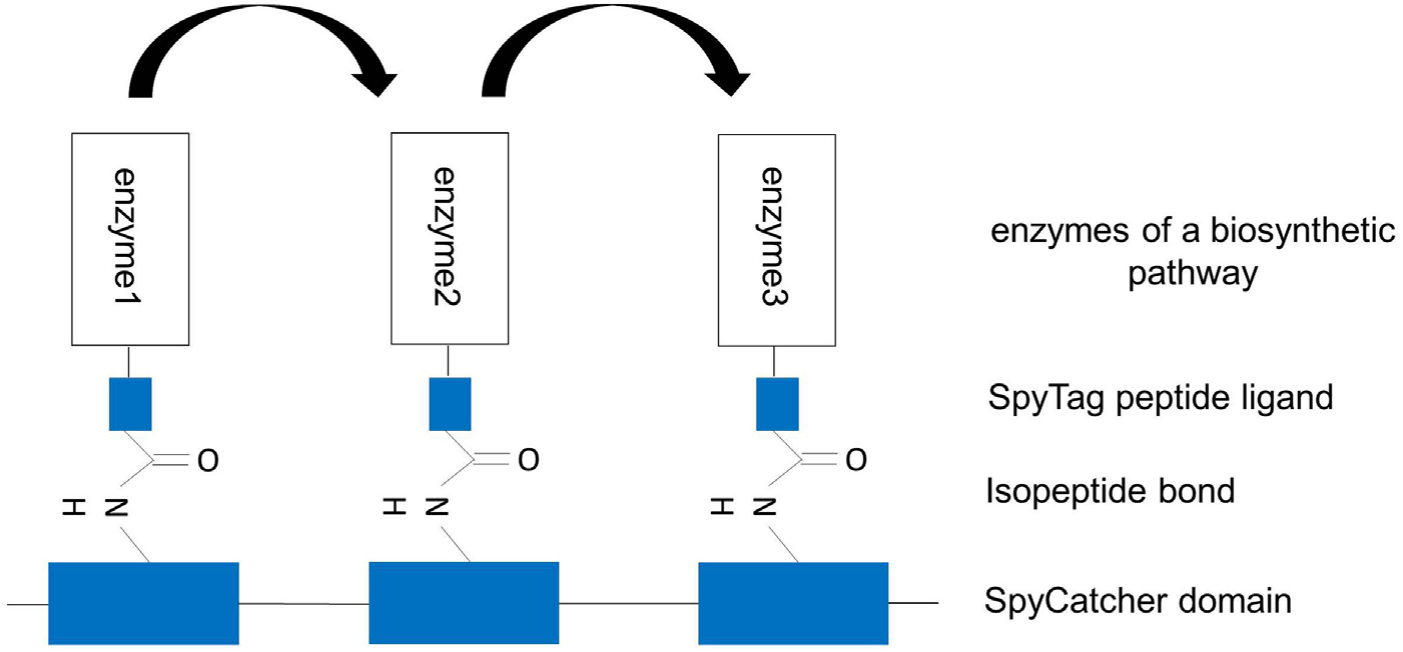
**Artificial multienzyme complex by covalently tethering pathway enzymes to the synthetic scaffold via the SpySystem**. The protein-based scaffold is based on repeats of SpyCatcher domains. The enzymes to be co-localized are fused to SpyTag peptide ligands. By co-expressing the components, the enzymes were co-immobilized on the scaffold via the covalent, irreversible interaction between SpyCatcher and SpyTag (isopeptide bond). Metabolic channeling between the single enzymes (indicated by the arrows) should be facilitated.

**Figure 6:**
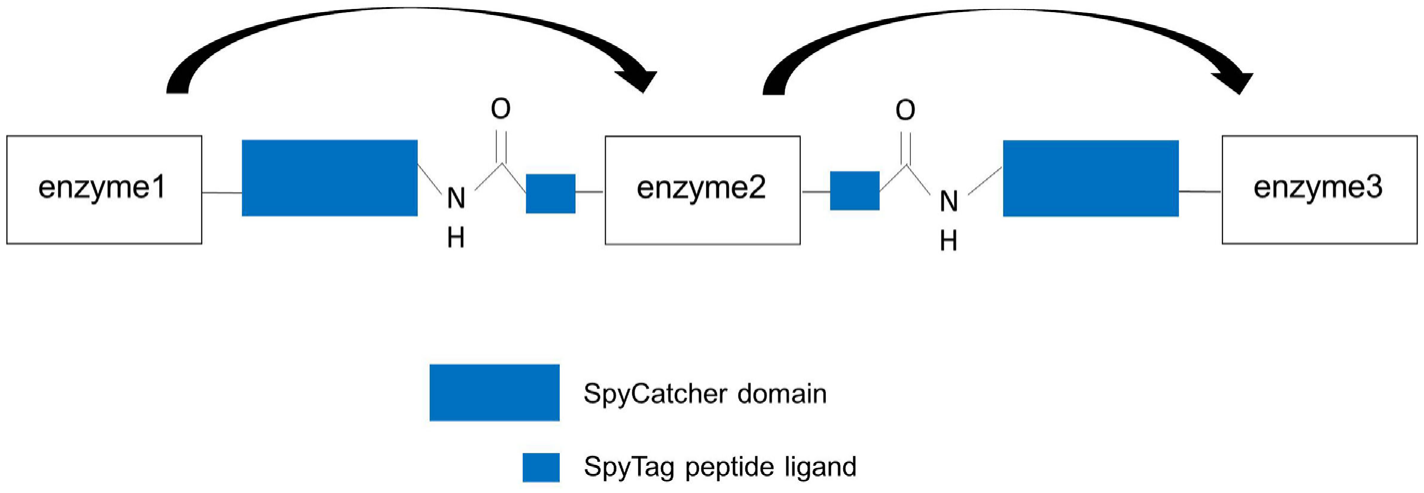
**Design of artificial trimeric proteins (functional trimer or even oligomers) by using the covalent SpySystem**. By mixing SpyCatcher-, respectively, SpyTag-modified enzymes artificial, covalent enzyme pipes can be generated. Linker sequences between the enzymes and the Spy-domains should ensure correct protein folding and structure. Metabolic channeling between the single, covalently linked enzymes (indicated by the arrows) should be facilitated.

The covalent SpySystem, pioneered by the group of Howarth, is based on the CnaB2 domain (immunoglobulin-like collagen adhesion domain) of the extracellular surface protein FbaB (fibronectin-binding protein) from the Gram-positive bacteria Spy. Within this CnaB2 domain, an autocatalytic, spontaneous, intramolecular reaction between a reactive lysine residue and a reactive aspartic acid residue takes place. This covalent isopeptide bond between Lys^31^ and Asp^117^ is catalyzed by a third amino acid residue, Glu^77^. These three amino acids form a catalytic triad and are directly involved in the isopeptide bond formation. In nature, a lot of Gram-positive bacteria were discovered to form spontaneous, intramolecular isopeptide bonds within their surface proteins. As a consequence of the covalent bond within a single protein, the protein is connected to itself conferring thermal, proteolytic, and pH stability to the protein. Consequently, bacteria use this covalent intramolecular bond formation to stabilize their extracellular proteins that are, for example, essential for the penetration and invasion of the host cells. By splitting the CnaB2 domain, two protein partners, SpyTag and SpyCatcher, were generated (Zakeri et al., 2012). The SpyTag (13 amino acids) contains the reactive aspartic acid residue whereas the SpyCatcher (138 amino acids) harbors the reactive lysine residue and the catalytic glutamic acid residue. *In vitro* and *in vivo* experiments have shown that the two protein partners find each other, reconstitute and undergo covalent reaction simply upon mixing. The isopeptide bond forms within minutes and is stable under a wide range of conditions (pH, temperature, buffer composition, reducing agents, and detergents) (Zakeri et al., 2012). This fact is important and should be highlighted as other systems such as covalent disulfide bonds easily dissociate under reducing conditions and are therefore reversible (Veggiani et al., 2014). Due to the robustness and covalent character of this SpySystem and the fact that all components are genetically encoded and can be expressed efficiently in *E. coli*, the SpySystem is ideal for irreversibly attaching proteins to each other. By fusing either SpyTag or SpyCatcher to the enzymes of interest, the catalyst can be covalently linked to corresponding SpyTag- or SpyCatcher-modified carriers (Figure 5). The SpySystem can also be used to create artificial multienzyme complexes that are stable. A synthetic scaffold composed of repeats of the SpyCatcher domain and SpyTag-enzyme conjugates are needed to co-localize sequential pathway enzymes. The final ratio of enzymes bound to SpyCatcher scaffolds is determined by the ratio of soluble SpyTag-modified enzymes mixed with the SpyCatcher scaffold and can only be controlled by a defined input enzyme stoichiometry which might be a disadvantage of this covalent SpySystem. The artificial multienzyme complex forms spontaneously upon mixing SpyTag- and SpyCatcher-modified enzymes and scaffolds. The SpyTag and SpyCatcher domains find each other, reconstitute and form a covalent isopeptide bond. To provide more flexibility so that the SpyCatcher- and SpyTag domains can fold properly and can undergo the isopeptide bonding, specific linker sequences, such as glycine-serine linkers, are inserted between the enzymes and the SpyTag or SpyCatcher domains. As the SpyTag sequence is very small, the fusion to enzymes should be no problem regarding correct folding, structure, and function of the enzyme. The covalent SpyCatcher–SpyTag system also can be used to covalently immobilize sequential pathway enzymes on carriers such as magnetic beads. By co-localizing pathway enzymes on the same magnetic bead, the metabolic efficiency should be increased due to the enforced proximity of the enzymes and the favored enzyme-to-enzyme substrate channeling. In addition to the metabolic/kinetic benefits, the stability of the enzymes can be enhanced thanks to the co-immobilization. It is also feasible to create multifunctional proteins by fusing SpyTag sequences to the N- and C-terminus of a selected enzyme. Mixing this double tagged enzyme with two enzymes carrying one SpyCatcher each, trimeric complexes are possible (Figure 6).

## Are There Additional Systems Allowing Controlled Formation of Intermolecular Linkages in Proteins?

**Figure 7:**
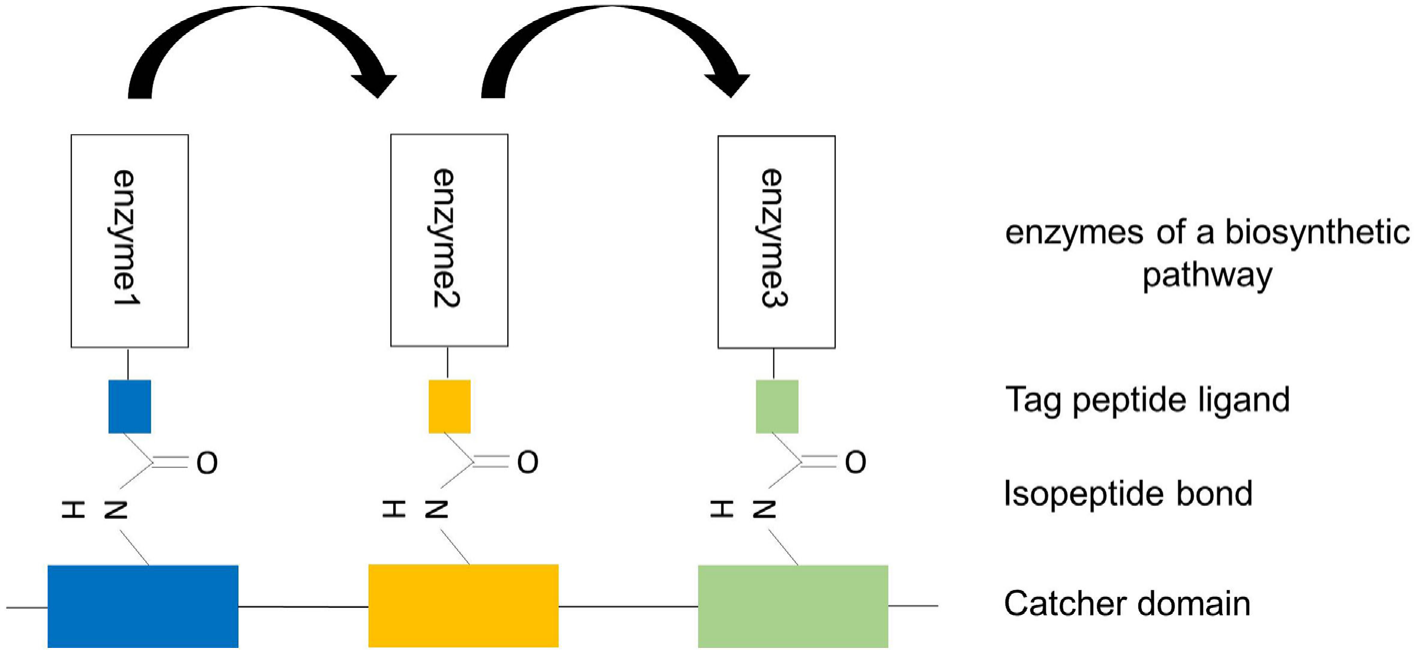
**Design of artificial trimeric proteins (functional trimer or even oligomers) by using different, covalent, alternative SpySystems**. The scaffold consists of different unique Catcher domains and the enzymes to be co-localized are fused to the corresponding specific Tag domains. Thanks to the specific and covalent interaction between the Catcher domains and their Tag domains, the enzymes are irreversibly targeted and bound to the scaffold. The specificity of this system enables the precise control of the enzyme ratio and the exact position of the enzymes at the scaffold. Metabolic channeling (indicated by the arrows) should be possible because of the enforced proximity of the sequential pathway enzymes.

To allow the design of precisely ordered enzyme arrays, alternative systems, in addition to the SpyTag-SpyCatcher system, for covalent tagging of proteins are required. Intermolecular isopeptide bonds are long known and form, for instance, between sumo or ubiquitin and lysine residues of target proteins. These modifications are linked to protein function, localization, and degradation. Intramolecular isopeptide bonds have only recently been discovered. They were first found in crystal structures of Spy0128 and have subsequently been detected in *Staphylococcus aureus* adhesin Can, *Enterococcus faecalis* adhesin Ace, *Streptococcus gordonii* antigen I/II adhesin SspB, and others (Kang and Baker, 2011). As intramolecular isopeptide bonds are frequently seen in extracellular proteins of Gram-positive bacteria, there is a good chance to find alternative covalent SpyCatcher–SpyTag interactions. If this search is successful, one can imagine that the targeting of Tag-enzyme conjugates to their specific Catcher binding domains is more specific and can be precisely controlled (Figure 7). As an alternative to isopeptide bonding, it may also be feasible to utilize the autocatalytic formation of thr-gln ester bonds as has been reported for the *Clostridium perfringens* adhesion protein Cpe0147 (Kwon et al., 2014). In a recent study of Gao et al. (2015) the authors combined non-covalent, high-affinity protein–protein interaction between the PDZ domain and its peptide ligand with covalent disulfide bonding. To this end, the authors substituted one amino acid in the PDZ domain and ligand by cysteine. Fusing these modified interaction domains to target proteins, the authors could demonstrate that depending on the redox potential of the buffer disulfide bridges could be formed between both domains. This disulfide bonding allowed the formation of “disulfide-locked multienzyme supramolecular devices” (Gao et al., 2015).

## 3-D Printing – A New Tool in Designing Enzyme Arrays

Three-dimensional printing is revolutionizing industry and holds great promise in biotechnology/biomedicine. Nowadays, it is possible to process a wide range of biological substances ranging from animal cells, plant cells, bacteria, proteins up to DNA sequences. Different strategies have been employed to transfer biological systems into micropattern, lines, channels, or other 3-D structures. These include photolithography, dip pen nanolithography, microcontact printing, replica molding, and AM eventually coupled with self-assembly techniques (Herzer et al., 2010). For example, photolithographic patterning of proteins on surfaces has been used extensively in the past to analyze cell behavior with micrometer-scale resolution (Kane et al., 1999). However, by photolithographic patterning it is hard depositing several different proteins on the same surface. Furthermore, in order to print microarrays in high-resolution structures down to approx. 100 nm, microcontact printing has been established as the method of choice. A 3-D shaped stamp transfers its surface layout onto a planar target structure (surface patterning). 3-D printing, as AM technique, adds the desired material in its final shape without subtractive removal. Historically, AM started first at an industrial level in the early 1990s as an alternative to traditional model-making techniques for rapid prototyping (sometimes referred to as 3-D printing). AM techniques are now defined by the American Society for Testing and Materials (ASTM) as processes of joining materials to make objects from 3-D digital data, usually layer upon layer, as opposed to subtractive manufacturing methods such as computer numerical control (CNC) milling (ASTM F2792-10 Standard Terminology for Additive Manufacturing Technologies, ASTM Intern., USA). Engineering of metabolic pathways is a highly complex and demanding approach, including large scale and high-throughput biology, manufacturing techniques with high resolution and enormous flexibility. Inkjet printing and bioplotting could be suitable AM techniques for a successful application in engineering of metabolic pathways.

**Figure 8:**
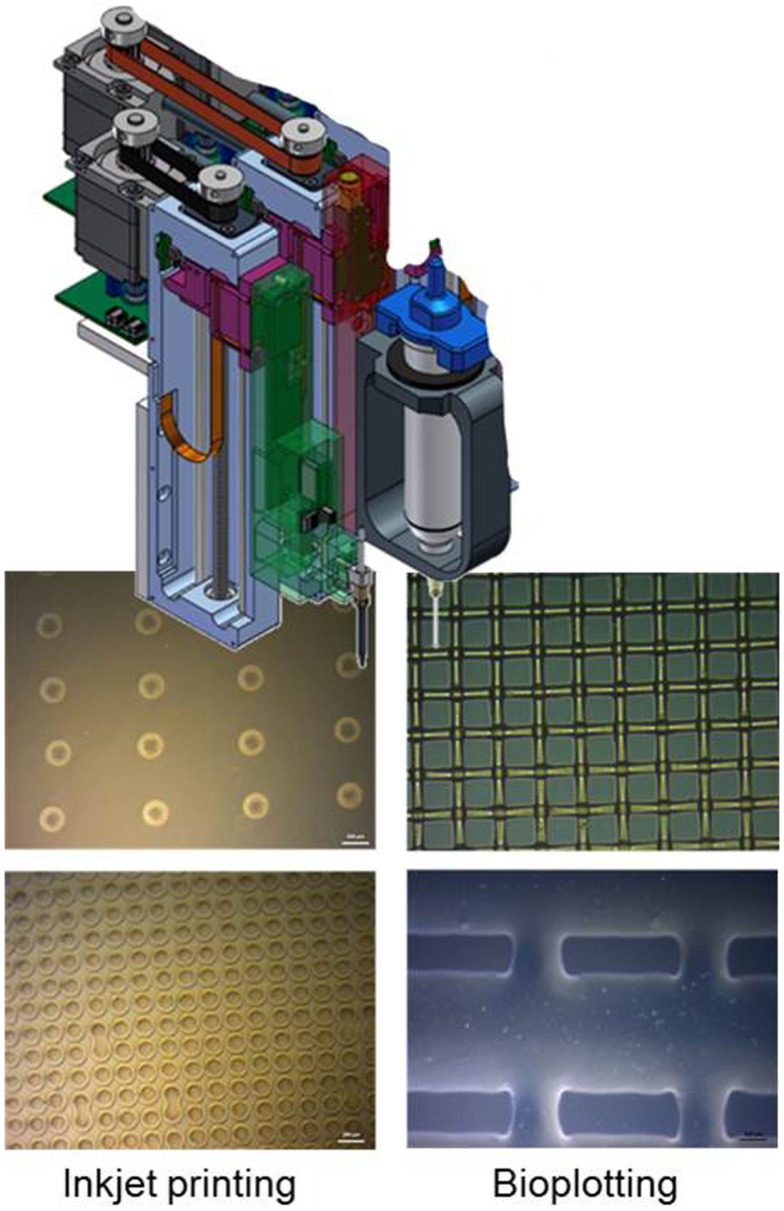
**Scheme of AM-technologies inkjet printing and bioplotting within light microscope images of typical generated dot and grid structures (Scale bar in all images 200 μm)**.

The inkjet technique is a non-contact digital printing method, creating a functional pattern by delivering ink droplets to the substrate surface. One type is the continuously injecting one, which shoots a permanent ink stream. The second one is the drop on demand (DOD) technique, which expulse only a single drop on a signal. The two main technics used are piezoelectric and electro-thermal, which are fast, cheap, digital controlled and simple methods for structured pattering of biomaterials, cells, and protein molecules (Calvert, 2001). Electro-thermal is the easiest and most used DOD technique, while piezoelectric is a milder printing technique. Inkjet printing as suitable AM method has been applied for the creation of a microenvironment for cells, proteins, and oligonucleotides by printing water-based matrices with spatially defined patterns (Barbulovic-Nad et al., 2006). Generally, inkjet systems enable the fast printing of small droplets or patterns with volumes in the range of 50–250 pl with a high resolution (50–100 μm) as illustrated in Figure 8. The bioplotting process is significantly slower and possesses a lower resolution compared to inkjet printing (also Figure 8). However, larger volumes of up to 10 nl can be processed so that the process is especially well suited for the handling of immobilized cells (Zehnder et al., 2015). With the aid of the 3-D bioplotter, larger and more complex scaffold geometries without the need for support structures can be realized, in contrast to the inkjet printer. The advantage of this method is the possibility to print cells, growth factors, and enzymes at the same time into precisely defined 3-D structure. By using a multi-nozzle bioplotter, it is possible to create scaffold structures consisting of several different materials and metabolic enzymes (Detsch et al., 2014).

## Conclusion

Engineering of metabolic pathways holds great promise to pave the way for green and sustainable chemistry. Recent success in Systems and Synthetic Biology opened a large number of possibilities to design more and more complex metabolic pathways *in vitro* and *in vivo*, not foreseen until recently. Thanks to structural analysis and intensive research in protein–protein interactions, it becomes feasible to organize artificial pathways in ordered and regulated arrays. Synthetic scaffolds offer a modular and highly flexible tool for rationally, post-translationally organizing/co-localizing multiple functionally related enzymes in a defined and controllable manner because the protein binding domain/peptide ligand interactions as well as the DNA–DNA/RNA–RNA, respectively, the DNA–Protein/RNA–Protein interactions are specific, adaptable, engineerable and can be theoretically applied to all metabolic pathways only depending on the developed ligand–enzyme conjugates or DNA/RNA–enzyme conjugates. So the main advantage of the scaffold-based multienzyme complex formation strategy is the modularity. The scaffold architecture and therefore the enzyme stoichiometry and enzyme ratio at the synthetic complex can be easily controlled just by varying single, modular biological parts. Through adding, removing, or replacing distinct domains, the scaffold structure is changed which can lead to a completely new behavior of the immobilized components and thereby of the whole pathway. Strategies for reversible interactions between proteins are complemented by covalent bonding enabling the stable construction of large multienzyme complexes. In addition to advances in the field of protein chemistry, technical developments such as 3-D printing will enable the design of industrial scale metabolic channels in the near future.

## Author Contributions

All authors contributed to writing and providing the literature, which has been cited in this review article.

## Conflict of Interest Statement

The authors declare that the research was conducted in the absence of any commercial or financial relationships that could be construed as a potential conflict of interest.
